# Psychometric properties of the Flourishing Scale for South African first-year students

**DOI:** 10.4102/ajopa.v5i0.130

**Published:** 2023-03-24

**Authors:** Karina Mostert, Leon T. de Beer, Ronalda de Beer

**Affiliations:** 1 Management Cybernetics Research Entity, Faculty of Economic and Management Sciences, North-West University, Potchefstroom, South Africa; 2 WorkWell Research Unit, Faculty of Economic and Management Sciences, North-West University, Potchefstroom, South Africa

**Keywords:** flourishing, factorial validity, item bias, differential item functioning, measurement invariance, internal consistency, first-year students, university

## Abstract

This study focused on a positive construct of well-being, namely flourishing. In a multicultural and diverse country such as South Africa, it is a legal requirement to provide evidence that measures of psychological constructs, like flourishing, are fair, unbiased, and equivalent for diverse groups in the country. The aim was to test the psychometric properties of the Flourishing Scale, a purpose-made scale that measures positive functioning across various areas of life. This study tested the factorial validity, item bias, measurement invariance and reliability of the Flourishing Scale in a sample of 1088 South African first-year university students. A unidimensional structure was confirmed. Although three items showed statistically significant uniform and total bias for language and campus groups, the magnitude and practical impact were negligible. No evidence of bias across gender groups was found. Configural, metric and partial scalar invariance were established for language and campus groups. Full measurement invariance was established across gender groups. Cronbach’s alpha coefficient was 0.91, indicating high reliability. The study provided promising results for using the Flourishing Scale among South African university students to measure flourishing as an aspect of well-being.

**Contribution:**

This study contributes to the field of student well-being in South Africa. No studies could be found that test for item bias or measurement invariance of the Flourishing Scale, specifically for South African first-year students. This study is the first to test these psychometric properties of a Flourishing Scale in a multicultural setting for students from different languages.

## Introduction

It is well established that first-year students face various challenges when transitioning from secondary to tertiary education (Kelly & Finlayson 2016; Nair & Fisher 2000; Van Zyl 2016). As students are often far from their loved ones, they feel alone, isolated, and stressed (Eagan et al. 2015). As a result, transitioning to higher education and adjusting to all the unfamiliar challenges encountered during the first year can negatively affect students’ well-being (Eagan et al. 2015; Vuckovic, Riley & Floyd 2019). However, it is also essential to identify and support students who are doing well and provide resources to help them flourish. The idea of flourishing has emerged as a critical component of subjective well-being (Diener et al. 2010). High levels of positive feelings characterise flourishing – the sense that one has a purpose in life, fosters positive relationships with others, cultivates optimism and strengthens high self-esteem (Diener et al. 2010). Flourishing also refers to a person’s knowledge of their life or how well they believe it to be and is linked to hedonic and eudemonic well-being (Keyes 2002).

Knowledge of students’ levels of flourishing could help Higher Education Institutions (HEIs) to motivate students to make an effort to achieve their academic objectives, enhance their welfare, and help train productive employees (Botha, Mostert & Jacobs 2019). The flourishing of first-year students is essential to HEIs, as this affects the process of graduation and their readiness to work (Jayawickreme & Dahill-Brown 2016; Schneiderman, Ironson & Siegel 2005).

Diener et al. (2010) developed a psychometric scale, the Flourishing Scale, to answer the need for a purpose-made scale to measure psychological flourishing. Although the scale does not give distinct metrics of different aspects of flourishing, it provides an overview of positive functioning across various areas in life generally perceived to be significant. The scale measures universal human psychological needs, meaning and purpose in life, optimism, and feelings of competence (Diener et al. 2010). This scale can be a valuable tool for HEIs to identify students’ flourishing levels to develop effective interventions to enhance levels of understanding and learn from students who are doing well at university who are thriving.

It is crucial to use scales that prove to be psychometrically sound. In a multicultural and diverse country such as South Africa, it is vital to test measures of psychological constructs to ensure they are fair, unbiased and equivalent for all ethnicities, languages, and other diverse groups in South Africa. South African law requires evidence that tests are appropriate, impartial and unbiased. This is stipulated in the *Employment Equity Act 55 of 1998*, Section 8 (Government Gazette 1998), which states that any form of psychological tests or similar assessments are prohibited unless the test or assessment being used is valid and reliable, can be applied fairly to all employees, and is not biased or discriminating against any employee or group.

The more rigorous testing of measures in diverse contexts are, it is not only applicable to South Africa, but also to other countries with diverse student populations. With the increasing migration and globalisation, many countries have become more diverse and multicultural (Van De Vijver & Rothmann 2004). It is also true for HEIs, where there is an influx of international students who need support (McKay, O’Neill & Petrakieva 2018). Multicultural testing is therefore of interest to other diverse settings, including student populations.

Central to multicultural assessment is bias and equivalence concepts (Van De Vijver & Rothmann 2004). Bias refers to certain nuisance factors that impede the comparability of test scores. Equivalence testing ensures the comparability of test scores across cultures or groups. When test scores are free of bias and demonstrate equivalence (or invariance), the scores can be compared across cultures or different sub-groups. Of particular interest are item bias and measurement invariance. Item bias (also referred to as differential item functioning [DIF]) occurs when respondents from different groups score differently on the item, even though they have the same standing on the underlying construct. Familiar sources of item bias include: differential response styles, poor item translation and ambiguous items, and the connotative meaning and appropriateness of the item content based on cultural specifics. Measurement invariance has: (1) configural invariance (the extent to which a factor structure can be replicated across groups), (2) metric invariance (equal factor loadings for similar items across groups), and (3) scalar invariance (similar meaning or interpretation for different groups) (Laher 2008; Van De Vijver & Rothmann 2004).

In addition, confirmatory factor analysis (CFA) and internal consistency (Cronbach’s coefficient alpha) were used to test the factor structure and reliability of the Flourishing Scale. Concerning factorial validity, the scale has a one-factor structure (Didino et al. 2019; Duan & Xie 2019; Muñoz & Nieto 2019; Singh, Junnarkar & Jaswal 2016), also in student samples (Hone, Jarden & Schofield 2014; Senol-Durak & Durak 2019; Sumi 2014). Many studies have shown that the Flourishing Scale has a high level of *internal consistency*, with Cronbach’s alpha coefficients ranging from 0.80 to 0.91 (Choudhry et al. 2018; Didino et al. 2019; Muñoz & Nieto 2019; Singh et al. 2016).

No studies could be found that test for item bias or measurement invariance of the Flourishing Scale, specifically for South African first-year students. Therefore, this study aims to provide psychometric evidence for the applicability of the Flourishing Scale in the diverse context of a South African university. More specifically, this study tested the factorial validity, item bias, metric, scalar and configural invariance, and internal consistency of the scale among first-year university students.

## Methods

### Participants

The study’s target demographic group was first-year university students enrolled at a South African university. A sample of 1088 participants was used, of which 72.4% were between the ages of 17 and 20 years and 16.7% were between 21 and 22 years. South Africa has 11 official languages distributed in different parts of the country. The languages most frequently used by students of the participating university were included in the analyses: Afrikaans (260, 23.9%), Setswana (199, 18.3), Sesotho (152, 14.0%) and English (94, 8.6%). The university has three campuses: Campus 1 is a campus located in a peri-urban area (131, 12%), Campus 2 is located in a medium-sized urban city (478, 43%), and Campus 3 is a smaller campus located in a large industrial city. In total, 689 (63.3%) females and 319 (29.3%) males participated in the study. Most participants were black students (62.3%), followed by white students (22.2%).

### Instrument

The Flourishing Scale (Diener et al. 2010) is a concise eight-item measure of respondents’ self-perceived performance in critical life domains such as relationships, self-esteem, intention, and optimism. A 7-point Likert scale was used, ranging from 1 (strongly disagree) to 7 (strongly agree). An example item is: ‘I lead a purposeful and meaningful life’. A high score indicates that the individual possesses psychological resources and strengths. The scale showed good psychometric qualities. The Cronbach’s alpha coefficient is reported as 0.82 (Diener et al. 2010).

### Procedure

The participating university accepted and authorised the project, and the study was granted ethics clearance. A secure direct link to the questionnaire was put on the university’s online portal. Throughout the study’s duration, students were informed about the research and encouraged to participate voluntarily. This was accomplished through field workers who presented brief awareness sessions in classrooms. Before completing the questionnaire, participants were required to sign an informed consent form. Furthermore, participants were assured that their reported responses would be anonymous, that the data gathered in the study would adhere to the project’s confidentiality criteria, and that the findings would be carefully stored in a secure database that would be password protected.

### Data analysis

MPlus 8.6 (Muthén & Muthén 2021) was used to conduct the statistical analyses. Confirmatory factor analysis was used to test the factorial validity of the Flourishing Scale. Maximum likelihood estimation was used, with the covariance matrix as input. The following fit indices were considered to assess the fit of the measurement model: the χ² statistic, the comparative fit index (CFI), the Tucker–Lewis index (TLI), the root mean square error of approximation (RMSEA), and the standardised root mean square residual (SRMR). Proper fit is considered at a value of 0.90 and above for the CFI and TLI (Byrne 2001; Hoyle 1995). For the RMSEA, a value of 0.05 or less indicates a good fit, whereas values between 0.05 and 0.08 are considered an acceptable model fit (Browne & Cudeck 1993; Chen et al. 2008).

Differential item functioning was used to test for the presence of item bias for language (four of the languages most frequently used by students at the participating university: Afrikaans, Setswana, Sesotho and English), campus (the three campuses described here) and also included males and females. Two forms of bias were tested: uniform and non-uniform bias. Uniform bias refers to the systematic difference in ability levels of the underlying construct between compared groups (Swaminathan & Rogers 1990; Teresi & Fleishman 2007). Non-uniform bias is the difference in the likelihood of related answers across different groups fluctuating across all ability levels (Swaminathan & Rogers 1990; Teresi & Fleishman 2007). The *lordif* package (Choi, Gibbons & Crane 2011) in RStudio Team (2020) was used. The following formulas were used and compared with test for uniform and non-uniform bias, using ordinal logistic regression to generate three likelihood-ratio χ² statistics (Choi et al. 2011):

$$\text{Model }0:\text{logit }P(u_{i}\geq k)=\alpha_{k}$$ [Eqn 1]

$$\text{Model }1:\text{logit }P(u_{i}\geq k)=\alpha_{k}+\beta_{1}*\text{ability}$$ [Eqn 2]

$$\text{Model }2:\text{logit }P(u_{i}\geq k)=\alpha_{k}+\beta_{1}*\text{ability}+\beta_{2}*\text{group}$$ [Eqn 3]

$$\begin{array}{l} \text{Model }3:\text{logit }P(u_{i}\geq k)=\alpha_{k}+\beta_{1}*\text{ability}+\\ \beta_{2}*\text{group}+\beta_{2}*\text{ability}*\text{group} \end{array}$$ [Eqn 4]

Biased items are flagged when statistically significant differences are detected, that is when the log-likelihood values of models are compared and *p* < 0.01; for uniform bias when comparing Models 1 and 2 $\left(\chi_{12}^{2};\text{degree of freedom }[df]=1\right)$, for non-uniform bias when comparing Models 2 and 3 $\left(\chi_{23}^{2};df=1\right)$; for a total DIF effect, comparing Models 1 and 3 $\left(\chi_{13}^{2};df=2\right)$ (Choi et al. 2011). The pseudo-Mcfadden *R*^2^ statistic is used to quantify the impact or practically significant effect of DIF, classifying the magnitude of DIF as negligible (< 0.13), moderate (between 0.13 and 0.26), or large (> 0.26) (Zumbo 1999). In addition, the impact of uniform DIF can be determined using the *β*_1_ coefficient when Models 1 and 2 are compared (Crane, Van Belle & Larson 2004). Different thresholds, ranging from a 10% difference between Models 1 and 2, indicate a practically meaningful effect (Crane et al. 2004; Maldonado & Greenland 1993).

Measurement invariance was investigated for the same language, campus, and gender groups. This was carried out in a multigroup analysis framework including the: (1) configural invariance model (i.e. the baseline model for the more constrained models and the test if a similar underlying latent factor is evident in the different groups); (2) metric invariance model (assumes the invariance or similarity of the factor loading in the different groups); and (3) scalar invariance model (test if the factor loadings and item intercepts are invariant or similar in the different groups) (Preti et al. 2013). The CFI and RMSEA values were used. For CFI, the fit is considered adequate if values are > 0.90 and better if they are > 0.95. For RMSEA, the cut-off value is < 0.08, but better is < 0.05 (Van De Schoot, Lugtig & Hox 2012). In addition, changes in CFI were used as recommended by Shi et al. (2019). A ΔCFI value higher than 0.01 between two nested models indicates that the added group constraints have led to a poorer fit; in other words, the more constrained model is rejected. By freeing the loading of items, partial metric invariance can be achieved (Cheung & Rensvold 2002; Preti et al. 2013). Cronbach’s alpha coefficient was used to determine the reliability of the scales. A cut-off point of 0.70 is deemed satisfactory (Nunnally & Bernstein 1994).

### Ethical considerations

The study was approved by the Ethics Committee, Faculty of Economic and Management Sciences (EC-EMS) (Ethics no.: NWU-HS-2014-0165-A4). Before completing the questionnaire, participants were required to sign an informed consent form. In addition, participants were assured that their reported responses would be anonymous, that the data gathered in the study would adhere to the project’s confidentiality criteria, and that the findings would be stored in a secure database that is password protected.

## Results

### Factorial validity

With regard to the factorial validity of the Flourishing Scale, a one-factor structure showed a good fit to the data (χ^2^ = 180.11; *df* = 19; CFI = 0.94; TLI = 0.91; RMSEA = 0.079; SRMR = 0.04). The standardised loadings are shown in Table 1.

**TABLE 1:** Standardised factor loadings.

Item	Loading	s.e.	*p*
Item 1	0.80	0.017	0.001
Item 2	0.74	0.025	0.001
Item 3	0.73	0.023	0.001
Item 4	0.77	0.019	0.001
Item 5	0.78	0.021	0.001
Item 6	0.74	0.022	0.001
Item 7	0.74	0.022	0.001
Item 8	0.65	0.023	0.001

Note: All *p*-values < 0.001.

s.e., standard error.

All items had high factor loadings (λ) (Shevlin et al. 1998), ranging from 0.65 (Item 8) to 0.80 (Item 1).

### Item bias (differential item functioning)

Uniform, non-uniform and total bias were tested (see Table 2).

**TABLE 2:** Differential item functioning.

Group	Item	$\chi_{12}^{2}u$	$\chi_{13}^{2}t$	$\chi_{23}^{2}n$	Δ*β*_1_	$R_{12}^{2}$	$R_{13}^{2}$	$R_{23}^{2}$
Language	Item 1	0.9674	0.8843	0.5529	0.0009	0.0001	0.0013	0.0011
	Item 2	**0.0005**	**0.0001**	0.0249	0.0130	0.0086	0.0131	0.0045
	Item 3	**0.0000**	**0.0000**	0.0100	0.0155	0.0118	0.0177	0.0059
	Item 4	0.3427	0.4710	0.5220	0.0080	0.0018	0.0029	0.0012
	Item 5	0.2457	0.1924	0.2096	0.0036	0.0021	0.0043	0.0022
	Item 6	0.0319	0.0326	0.1762	0.0102	0.0046	0.0072	0.0026
	Item 7	**0.0000**	**0.0000**	0.2685	0.0513	0.0201	0.0223	0.0021
	Item 8	0.6897	0.6853	0.4810	0.0028	0.0007	0.0019	0.0012
Campus	Item 1	0.8863	0.8623	0.5906	0.0005	0.0001	0.0005	0.0004
	Item 2	**0.0000**	**0.0000**	0.0406	0.0201	0.0071	0.0092	0.0021
	Item 3	**0.0000**	**0.0000**	0.0624	0.0376	0.0144	0.0162	0.0018
	Item 4	0.4688	0.2599	0.1523	0.0030	0.0005	0.0019	0.0013
	Item 5	0.8188	0.2571	0.0867	0.0013	0.0001	0.0019	0.0018
	Item 6	0.2490	0.5541	0.8863	0.0007	0.0010	0.0011	0.0001
	Item 7	**0.0000**	**0.0000**	0.3132	0.0088	0.0086	0.0095	0.0009
	Item 8	0.8647	0.4007	0.1534	0.0010	0.0001	0.0014	0.0013
Gender	Item 1	0.0699	0.1597	0.5352	0.0072	0.0011	0.0013	0.0001
	Item 2	0.6454	0.2671	0.1192	0.0020	0.0001	0.0008	0.0008
	Item 3	0.0612	0.1404	0.5159	0.0076	0.0011	0.0012	0.0001
	Item 4	0.5433	0.4923	0.3061	0.0018	0.0001	0.0005	0.0003
	Item 5	0.9806	0.7361	0.4340	0.0001	0.0000	0.0002	0.0002
	Item 6	0.0391	0.1164	0.8322	0.0029	0.0014	0.0014	0.0000
	Item 7	0.5946	0.6462	0.4424	0.0013	0.0001	0.0003	0.0002
	Item 8	0.0475	0.0881	0.3344	0.0045	0.0013	0.0016	0.0003

Notes: $\chi_{12}^{2}$, chi-square of model 1 compared with model 2; $\chi_{13}^{2}$, chi-square of model 1 compared with model 3; $\chi_{23}^{2}$, chi-square of model 2 compared with model 3; *β*_1_, change in beta coefficient; $R_{12}^{2}$, pseudo-Mcfadden *R*^2^ of model 1 compared with model 2; $R_{13}^{2}$, pseudo-Mcfadden *R*^2^ of model 1 compared with model 3; $R_{23}^{2}$, pseudo-Mcfadden *R*^2^ of model 2 compared with model 3. Values in bold text are when statistically significant differences are detected (*p* < 0.01) when the log-likelihood values of models are compared.

**FIGURE 1:**
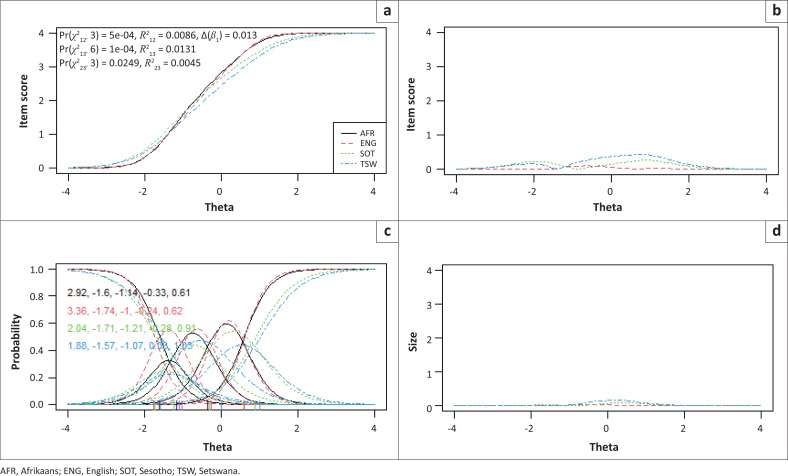
Graphical display of Item 2, which shows uniform and non-uniform differential item functioning with respect to language groups. (a) Items True Score Functions - item 2; (b) differences in items True Score Functions; (c) Item Response Functions; (d) Impact (weighed by density).

**FIGURE 2:**
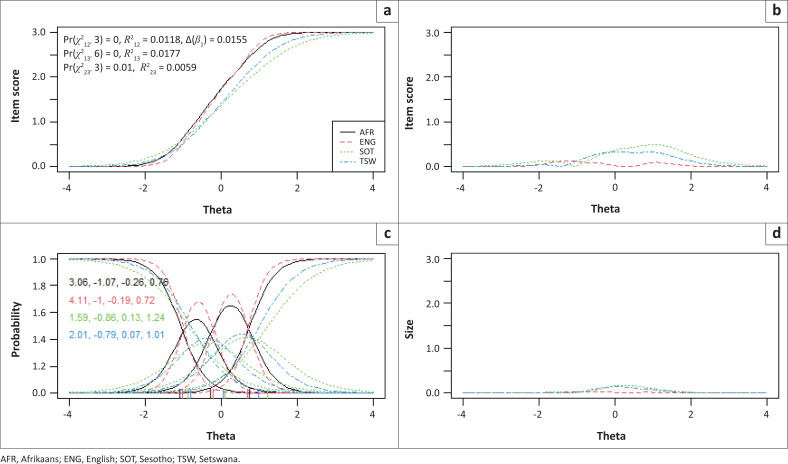
Graphical display of Item 3, which shows uniform and non-uniform differential item functioning with respect to language groups. (a) Items True Score Functions - item 3; (b) differences in items True Score Functions; (c) Item Response Functions; (d) Impact (weighed by density).

**FIGURE 3:**
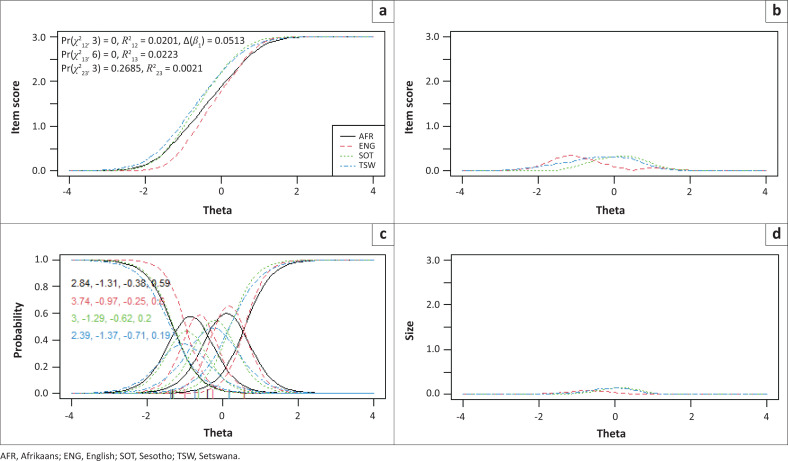
Graphical display of Item 7, which shows uniform and non-uniform differential item functioning with respect to language groups. (a) Items True Score Functions - item 7; (b) differences in items True Score Functions; (c) Item Response Functions; (d) Impact (weighed by density).

**FIGURE 4:**
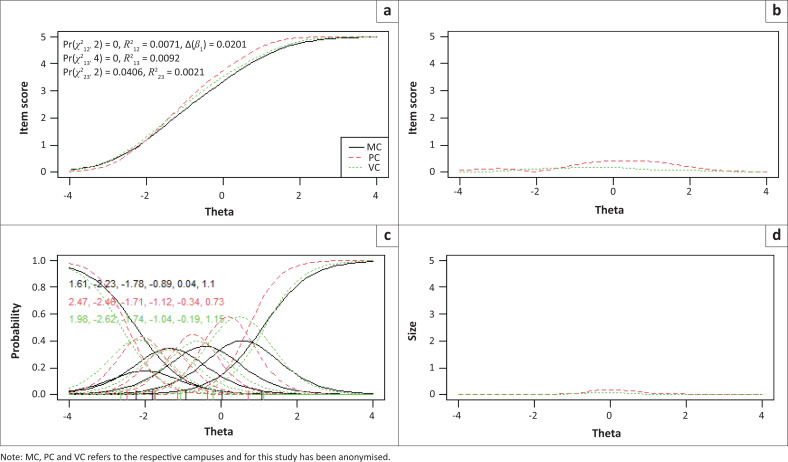
Graphical display of Item 2, which shows uniform and non-uniform differential item functioning with respect to campuses. (a) Items True Score Functions - item 2; (b) differences in items True Score Functions; (c) Item Response Functions; (d) Impact (weighed by density).

**FIGURE 5:**
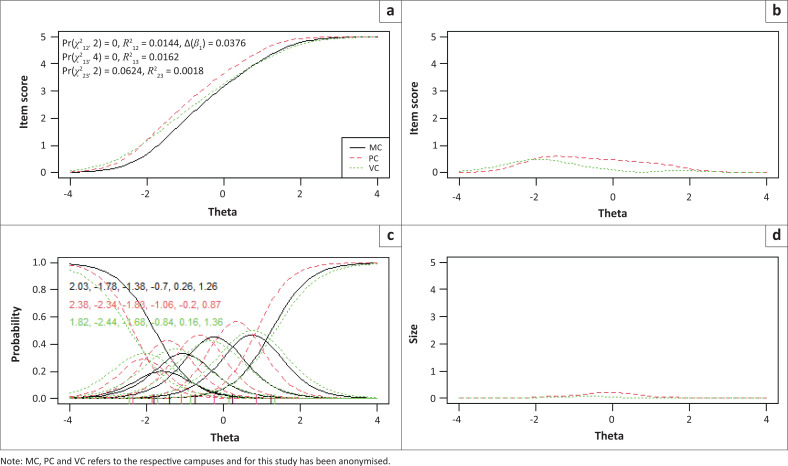
Graphical display of Item 3, which shows uniform and non-uniform differential item functioning with respect to campuses. (a) Items True Score Functions - item 3; (b) differences in items True Score Functions; (c) Item Response Functions; (d) Impact (weighed by density).

**FIGURE 6:**
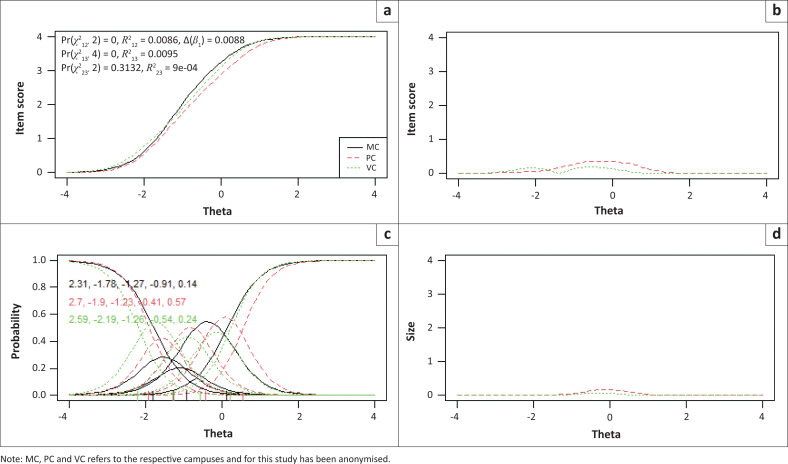
Graphical display of Item 7, which shows uniform and non-uniform differential item functioning with respect to campuses. (a) Items True Score Functions - item 7; (b) differences in items True Score Functions; (c) Item Response Functions; (d) Impact (weighed by density).

Items 2, 3 and 7 showed statistically significantly uniform and total bias for the included language and campus groups, while no bias was detected between males and females. To determine if the magnitude of DIF for these three items were of practical significance, pseudo-McFadden *R*^2^ values and the difference in the *β*1 coefficient were inspected. In addition, visual graphs are provided for each item to demonstrate the effect between language and campus groups (Figure 1, Figure 2, Figure 3, Figure 4, Figure 5 and Figure 6). Each of these figures present four graphs providing additional diagnostic information, including the item characteristic curve for the different groups (in this case, language and campus groups; upper-left graph); the item response functions for the parameter estimates for each group (lower-left graph); the absolute difference between item characteristic curves for sub-groups (upper-right graph); and the absolute difference between the item characteristic curves of the sub-groups weighted by the score distribution (Choi et al. 2011).

For all three items in language and campus groups, the differences between language and campus groups were slightly different compared with each other; however, these differences were negligible, as can be seen in the density-weighted impact in each figure (bottom right plots). Also, the pseudo-McFadden *R*^2^ statistic values were all smaller than 0.13 and the difference in *β*_1_ coefficients smaller than 5%. As a result, DIF’s magnitude or practical impact on these three items can be classified as negligible.

### Measurement invariance

The results of the configural, metric and scalar invariance testing across the language, campus, and gender groups included in this study are shown in Table 3.

**TABLE 3:** Measurement invariance analysis.

Variable	χ^2^	*df*	CFI	Δ CFI	RMSEA	Δ RMSEA
**Language**						
Configural	195.26	76	0.939	-	0.094	-
Metric	230.06	97	0.932	−0.007	0.088	−0.006
Scalar	297.10	118	0.908	−0.024	0.093	0.005
Partial scalar	260.91	114	0.925	−0.007	0.086	−0.002
**Campus**						
Configural	251.09	57	0.927	-	0.103	-
Metric	281.10	71	0.921	−0.006	0.096	−0.007
Scalar	342.27	85	0.903	−0.018	0.097	−0.006
Partial scalar	307.09	81	0.915	−0.006	0.093	−0.003
**Gender**						
Configural	215.73	38	0.930	-	0.096	-
Metric	233.68	45	0.926	−0.004	0.091	−0.005
Scalar	257.84	52	0.919	−0.007	0.089	−0.002

*χ*^2^, Chi-square; df, degrees of freedom; CFI, comparative fit index; ΔCFI, delta (change in) CFI; RMSEA, root mean square error of approximation; ΔRMSEA, delta (change in) RMSEA.

With regard to language and campus, configural and metric invariance were established. The results of scalar invariance showed that ΔCFI for language was –0.024 and for campus –0.018 (higher than 0.01). Consequently, partial scalar invariance was established, releasing the intercept of items 4 and 7 in the Afrikaans and English language groups and items 3 and 7 in all three campus groups. Configural, metric and scalar invariance was confirmed for gender.

### Internal consistency

As a measure of internal consistency, Cronbach’s alpha coefficient was calculated to establish the internal consistency of the Flourishing Scale. With α = 0.91, the Flourishing Scale was found to be reliable (Nunnally & Bernstein 1994).

## Discussion

This study aimed to test the psychometric properties of the Flourishing Scale to determine if this scale is valid and reliable for assessing flourishing, a positive construct of psychological well-being, in South African first-year university students. The study’s primary objective was to determine the factorial validity, item bias, metric, scalar and structural invariance, and internal consistency.

Concerning the factorial validity, the results showed that a one-factor structure was a good fit for the data. The findings are consistent with previous studies, where a one-factor structure was confirmed in student samples from New Zealand, Turkey, and Japan (Hone et al. 2014; Senol-Durak & Durak 2019; Sumi 2014).

Differential item functioning was used to determine uniform and non-uniform bias. Statistically significant uniform and total bias were found across language and campus groups for items 2, 3 and 7. However, the magnitude or practical impact of this bias was negligible. This means that, on a practical level, the language, campus, and gender sub-groups included in this study understood the items identically across groups, and that no incongruities at the item level exist for participants in these sub-groups (Cleary & Hilton 1968; Van De Vijver & Tanzer 2004).

Regarding measurement invariance, configural invariance was established for all included sub-groups. The results show that the one-factor structure of the Flourishing Scale has the same pattern and fits the data equally well in all groups. Therefore, the factor structure can be replicated similarly for different language, campus and gender groups (Byrne, Shavelson & Muthén 1989; Putnick & Bornstein 2016). Metric invariance was also established for all sub-groups, indicating that the loading of each item contributes equally to the latent construct of flourishing across the different groups. Although scalar invariance was confirmed for gender, only partial scalar invariance was established for language and campus groups because of the ΔCFI values higher than 0.01 (Cheung & Rensvold 2002; Preti et al. 2013). This implies that specific item intercepts were not equivalent between language and campus groups. As a result, the intercepts of items 4 and 7 of two language groups (i.e. Afrikaans and English) and items 3 and 7 in all three campus groups had to be released to establish partial invariance. Even though these parameters can vary across groups, valid inferences can still be made when at least two intercepts and factor loadings are equally constrained, which is in line with the findings of previous studies (Laguna et al. 2017; Van De Schoot et al. 2012).

The Cronbach’s alpha coefficient was calculated to determine the internal consistency of the Flourishing Scale and showed a reliability coefficient of 0.91. Various research studies have found that the Flourishing Scale has a high level of internal consistency, with Cronbach’s alpha coefficients ranging from 0.80 to 0.91 (Choudhry et al. 2018; Didino et al. 2019; Muñoz & Nieto 2019; Singh et al. 2016).

### Limitations and recommendations

Even though the findings of this study are promising, several limitations must be mentioned. The study’s primary focus was on first-year university students in South Africa. Therefore, the study should be replicated for senior students, other universities, and other countries with multicultural populations. South Africa has 11 official languages, of which only 4 were included in this study. Other language groups should also be included in future studies. Three items seemed to be somewhat problematic (items 3, 4 and 7) regarding bias and invariance. Even though the practical effect was small and negligible, future studies should investigate how these items function in other samples.

## Conclusion

This study provides initial support for using the Flourishing Scale in a South African sample of first-year university students and opens the way for its further use in other student samples. The scale demonstrated high reliability, and the DIF and invariance analyses confirmed that no practically significant incongruities exist between language, campus, and gender groups.
